# Endoscopic traversability in patients with locally advanced esophageal squamous cell carcinoma: Is it a significant prognostic factor?

**DOI:** 10.1097/MD.0000000000009441

**Published:** 2017-12-22

**Authors:** Hae Jin Shin, Hee Seok Moon, Sun Hyung Kang, Jae Kyu Sung, Hyun Yong Jeong, Seok Hyun Kim, Byung Seok Lee, Ju Seok Kim, Gee Young Yun

**Affiliations:** a Division of Gastroenterology, Department of Internal Medicine, Aerospace Medical Center, Republic of Korea Air Force, Cheongwon-gun, Chungcheongbuk-do; b Division of Gastroenterology, Department of Internal Medicine, Chungnam National University Hospital, Chungnam National University School of Medicine, Daejeon, Republic of Korea.

**Keywords:** definitive chemoradiotherapy, endoscopic traversability, esophageal cancer, malignant stricture, prognostic factor

## Abstract

The purpose of this study was to evaluate the prognostic impact of endoscopic traversability in patients with locally advanced esophageal squamous cell carcinoma.

This retrospective study was based on medical records from a single tertiary medical center. The records of 317 patients with esophageal squamous cell carcinoma treated with surgery or definitive chemoradiotherapy (CRT) between January 2009 and March 2016 were reviewed. Finally, we retrieved the data on 168 consecutive patients. These 168 patients were divided into 2 groups based on their endoscopic traversability findings: Group A (the endoscope traversable group), and Group B (the endoscope non-traversable group). We then retrospectively compared the clinical characteristics of these 2 groups.

The endoscope non-traversable group (Group B) revealed an advanced clinical stage, a poor Eastern Cooperative Oncology Group (ECOG) score, a lower serum albumin level, a higher rate of requirement for esophageal stent insertion and definitive CRT as initial treatment than the endoscope traversable group (Group A). Patients with endoscope traversable cancer showed a significantly higher 3-year overall survival and 3-year relapse-free survival than patients who were endoscope non-traversable (53.8% vs 17.3%, *P* < .001 and 71.1% vs 45.3%, *P* = .003, respectively). Upon multivariate analysis of patients with locally advanced esophageal squamous cell carcinoma treated with definitive CRT, the serum albumin level <3.5 g/dL and endoscopic non-traversability were significant negative factors of survival.

Endoscopic traversability in patients with locally advanced esophageal squamous cell carcinoma treated with definitive CRT is a significant prognostic factor.

## 1 Introduction

Malignant tumors arising in the esophagus are largely squamous cell carcinomas and adenocarcinomas. The 2 diseases differ from each other in their clinical progression, reaction to treatments, and prognosis; their causes are also substantially different.^[1]^ In the West, esophageal cancer is relatively rare, with a lifetime risk of <1%; in Korea, it is the seventh most common cancer, with squamous cell carcinoma being the predominant histopathologic type.^[2]^ Its prognosis is very poor; the 5-year overall survival rate is <20%.^[3,4]^ This high mortality rate can be attributed to the fact that 50% of patients have a locally advanced form of the disease at diagnosis, which is defined as having a tumor with periesophageal tissue invasion, contiguous structural involvement, or lymph node metastasis.^[5]^ In such cases, curative surgery can only be performed in 30% to 40% of patients.^[6]^

In localized esophageal cancers, the standard treatment is surgical resection, with endoscopic treatment being performed selectively in some early stage cases.^[7]^ However, when the cancer is diagnosed along with accompanying symptoms, metastasis to adjacent tissues via abundant submucosal lymphoid tissue is already advanced. Additionally, complete resection can be difficult around the trachea, tracheal bifurcation, and distal third of the esophagus due to the anatomy of these regions. Therefore, the frequency of systemic and local recurrences is notably high in locally advanced esophageal cancers even following complete tumor resection and lymph node dissection.

In many metastatic lesions, systemic chemotherapy or concomitant chemoradiotherapy (CRT) is often recommended instead of surgery.^[7]^ However, the prognosis remains poor compared with other gastrointestinal cancers; for metastatic esophageal cancer, the median overall survival time is still <6 months. Even if the cancer reacts positively to chemotherapy or concomitant CRT, this reaction is often transient.^[5]^

One of the major negative prognostic factors reported in patients receiving preoperative CRT and esophagectomy due to locally advanced esophageal squamous cell carcinoma is endoscopic ultrasonography (EUS) non-traversability.^[8]^ However, only limited studies have investigated the impact that conventional endoscopic traversability has on the prognosis in patients with locally advanced squamous cell carcinoma that have been treated with definitive CRT. Because esophagogastroduodenoscopy with biopsy plays an essential role in histologically confirming esophageal cancer, we questioned whether the prognosis of patients with esophageal squamous cell carcinoma can be easily predicted according to endoscopic traversability.

Therefore, the purpose of this study was to evaluate retrospectively the prognostic impact of endoscopic traversability on the overall survival and relapse-free survival in patients with locally advanced esophageal squamous cell carcinoma.

## 2 Materials and methods

### 2.1 Patients and tumor staging

**Figure 1:**
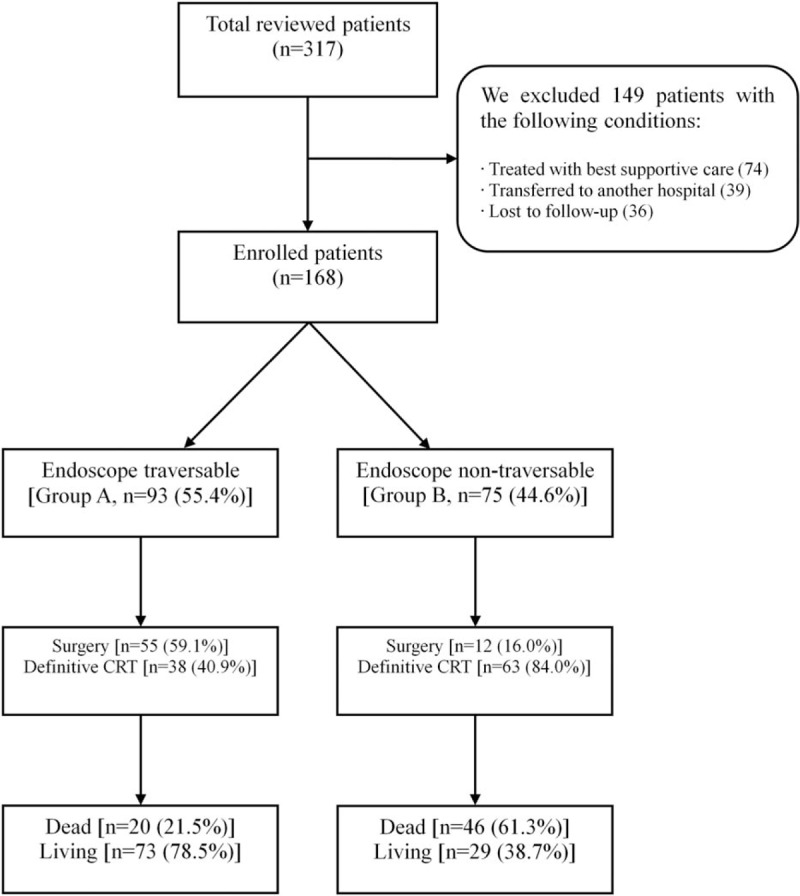
Flow chart of this study.

This was a retrospective study based on medical records from a Chungnam National University Hospital located in Daejeon, Republic of Korea. Between January 2009 and March 2016, 317 patients were diagnosed with esophageal squamous cell carcinoma. We excluded 149 of these from this study; 74 patients received the best supportive care, 39 were transferred to another hospital, and 36 patients were lost to follow-up. In total, 168 patients were enrolled in this study (Fig. 1). All the diagnoses of esophageal squamous cell carcinoma of patients included in this study were confirmed histologically and were all treated with first-line surgery or definitive CRT using radiotherapy with concomitant 5-fluorouracil (FU) plus cisplatin-based chemotherapy.

**Figure 2:**
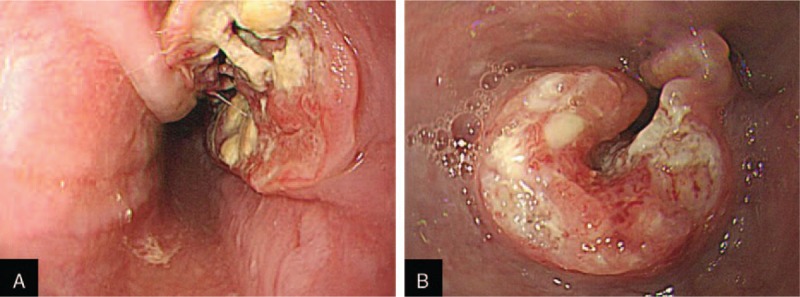
Endoscopic findings according to endoscopic traversability. (A) Endoscope traversable group findings, (B) endoscope non-traversable group findings.

The patients were divided into 2 groups according to endoscopic traversability: an endoscope traversable group (Group A) and an endoscope non-traversable group (Group B) (Fig. 2). Group A was comprised of patients with smooth entry without resistance using a conventional endoscope (GIF-H260: distal end outer diameter, 10.8 mm; Olympus, Tokyo, Japan). Group B included patients with severe resistance during entry using a conventional endoscope, and these patients had the following conditions: completely impossible entry with a conventional endoscope and accessible entry after changing to a pediatric endoscope (GIF-XP260: distal end outer diameter, 6.5 mm; Olympus, Tokyo, Japan). At the last follow-up visit, 20 of 93 patients (21.5%) in the endoscope traversable group and 46 of 75 patients (61.3%) in the endoscope non-traversable group had died.

Clinical and tumor baseline data were collected for each patient, and their performance status was evaluated according to the Eastern Cooperative Oncology Group (ECOG) score. Furthermore, the baseline nutritional status, which included the serum albumin level and body mass index (BMI), was also evaluated in each patient. In patients with severe malignant esophageal stricture, self-expandable metal stents were inserted for palliative measures.

All patients received esophagogastroduodenoscopy with biopsy, contrast-enhanced chest-abdominal computed tomography (CT), and 18F-fluorodeoxyglucose positron emission tomography-CT (PET-CT). Their clinical stages were determined using conventional imaging modalities, including contrast-enhanced chest-abdominal CT and PET-CT. Tumor stages were evaluated according to the AJCC TNM staging system. This study was approved by the Institutional Review Board of the Chungnam National University Hospital (IRB file number: CNUH 2016-12-024). For this retrospective study, written informed consent was not required.

### 2.2 Treatment plan (surgery and definitive CRT)

All patients underwent surgery or definitive CRT depending on their clinical stage. In all, 67 patients were treated with surgery (esophagectomy), which consisted of a transhiatal, abdominal-right thoracic (Ivor-Lewis), or right thoracic-abdominal-cervical (McKeown) approach. The proximal and distal margins from the gross esophageal tumor were required to be at least 6 to 8 cm. En bloc lymph node dissection included the paracardial, posterior mediastinal, infracarinal, and periesophageal lymph nodes.

In all, 101 patients were treated with definitive CRT. This method consisted of 5-FU plus cisplatin-based chemotherapy (a 75-mg/m^2^ bolus intravenous infusion of cisplatin was administered for 30 minutes on days 1 and 29, while 5-FU 1000 mg/m^2^ was given as a continuous intravenous infusion for 96 hours after completion of the cisplatin bolus intravenous infusion on days 1–4 and 29–32) with concurrent radiotherapy (50.4 Gy/23 fractions) over 4 weeks.

### 2.3 Follow-up evaluation and assessment of end points

During the follow-up period, the patients were assessed by clinical examinations, esophagogastroduodenoscopy, and contrast-enhanced chest-abdominal CT. Follow-up evaluations were carried out routinely every 3 months for the first year, every 6 months for the second year, and yearly thereafter. The patients were evaluated either until the cutoff date of this study, which was March of 2016, or until their deaths. Follow-up data were obtained from the patients’ medical records.

The end points for this study were the overall survival and the relapse-free survival times. The date of esophageal squamous cell carcinoma diagnosis was the starting point for the analysis of the overall survival and relapse-free survival.

### 2.4 Statistical analysis

Baseline clinical characteristics are expressed as a number (percentage) for categorical variables or as the means ± standard deviation (SD) for continuous variables. Categorical variables were compared using the Fisher exact test or chi-square test, and continuous variables were compared using the Student *t* test. The overall survival curves and relapse-free survival curves were determined by the Kaplan–Meier method and compared with the Log-rank test. Univariate and multivariate analyses along with Cox proportional hazards models were carried out to determine the predictive factors that influenced patient survival. All *P* values were 2-sided, and a *P* < .05 was considered statistically significant. All statistical analyses were performed using the Statistical Package for the Social Sciences (SPSS), version 21.0 (IBM Co., Armonk, NY).

## 3 Results

### 3.1 Patient characteristics and endoscopic traversability

Of the 168 patients considered eligible for this study, 93 (55.4%) were included in the endoscope traversable group (Group A), and the remaining 75 (44.6%) made up the endoscope non-traversable group (Group B). Table 1 presents the baseline clinical characteristics of these 2 groups. The median age of the study patients was 67.44 ± 8.44 years, and 160 patients were male.

**Table 1:**
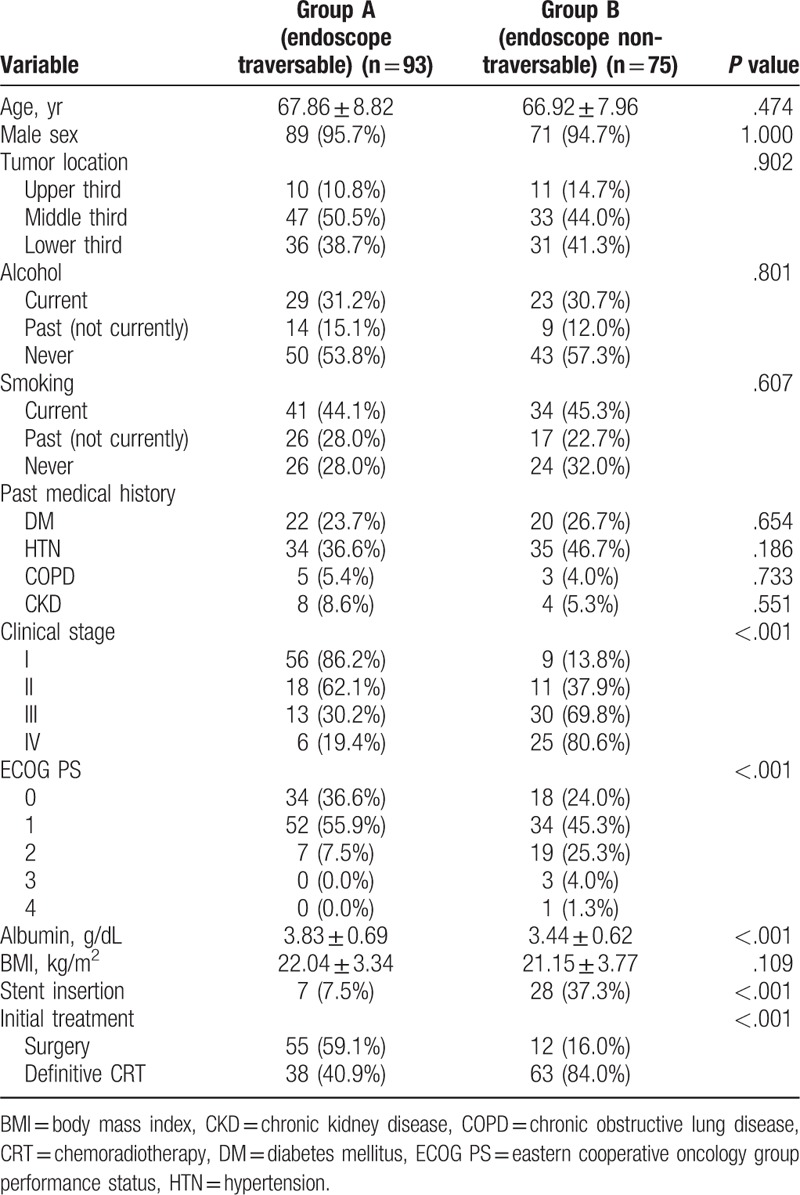
Baseline clinical characteristics of the study cohort.

The results showed significant clinical differences between the 2 groups. The endoscope non-traversable group (Group B) demonstrated an advanced clinical stage (*P* < .001), a poor ECOG performance status score (*P* < .001), a lower serum albumin level (3.83 ± 0.69 g/dL vs 3.44 ± 0.62 g/dL, *P* < .001), a higher rate of requirement for esophageal stent insertion (7.5% vs 37.3%, *P* < .001) and definitive CRT as initial treatment (40.9% vs 84.0%, *P* < .001) than the endoscope traversable group (Group A). Age, sex, tumor location, alcohol status, smoking status, past medical history, and BMI did not differ between the 2 groups.

### 3.2 Overall survival and relapse-free survival in all patients

**Figure 3:**
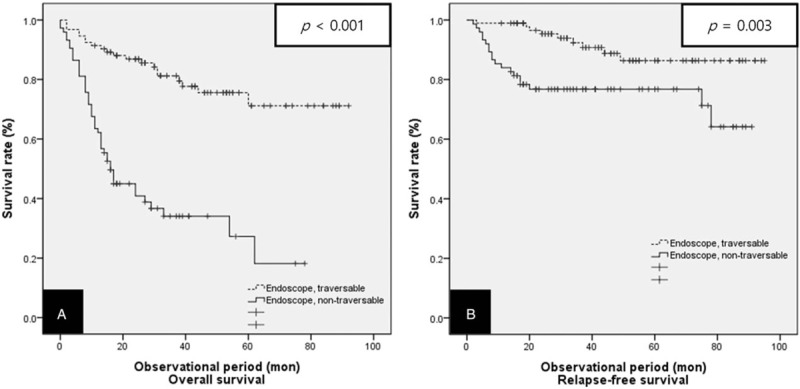
Kaplan–Meier curves for the overall survival and relapse-free survival in both groups of patients treated with surgery or definitive CRT. (A) Overall survival, (B) relapse-free survival. CRT = chemoradiotherapy.

After a median follow-up period of 26.50 months, the median overall survival time was 68.72 months (95% CI: 61.72–75.72 months) in the endoscope traversable group versus 28.36 months (95% CI: 21.53–35.19 months) for the endoscope non-traversable group. Patients who were endoscope traversable showed a significantly higher 3-year overall survival than the endoscope non-traversable patients (53.8% vs 17.3%, respectively, *P* < .001) (Fig. 3A). The median relapse-free survival time was 81.40 months (95% CI: 76.13–86.67 months) in Group A and 65.74 months (95% CI: 57.95–73.54 months) in Group B. Patients who were endoscope traversable showed a significantly higher 3-year relapse-free survival than patients in the endoscope non-traversable group (71.1% vs 45.3%, respectively, *P* = .003) (Fig. 3B).

### 3.3 Overall survival and relapse-free survival in patients treated with definitive CRT

**Figure 4:**
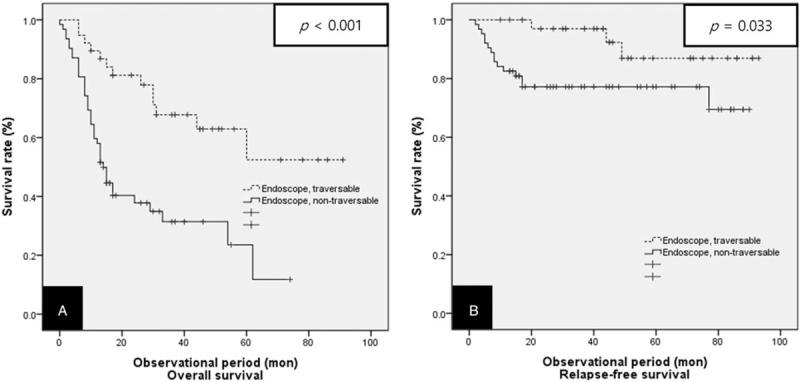
Kaplan–Meier curves for overall survival and relapse-free survival in both groups of patients treated with definitive CRT. (A) Overall survival, (B) relapse-free survival. CRT = chemoradiotherapy.

Of the 101 patients treated with definitive CRT, 38 (37.6%) were included in the endoscope traversable group (Group A), while the remaining 63 (62.4%) made up the endoscope non-traversable group (Group B). After a median follow-up period of 16.50 months, the median overall survival was 58.47 months (95% CI: 46.50–70.43 months) in the endoscope traversable group and only 25.76 months (95% CI: 18.93–32.59 months) in the endoscope non-traversable group. Patients in Group A showed a significantly higher 3-year overall survival than Group B patients (47.4% vs 15.9%, respectively, *P* < .001) (Fig. 4A). The median relapse-free survival time was 81.87 months (95% CI: 74.32–89.42 months) in the endoscope traversable group versus 66.87 months (95% CI: 58.45–75.29 months) in endoscope non-traversable patients. Group A showed a significantly higher 3-year relapse-free survival than patients Group B (71.1% vs 47.6%, respectively, *P* = .033) (Fig. 4B).

**Figure 5:**
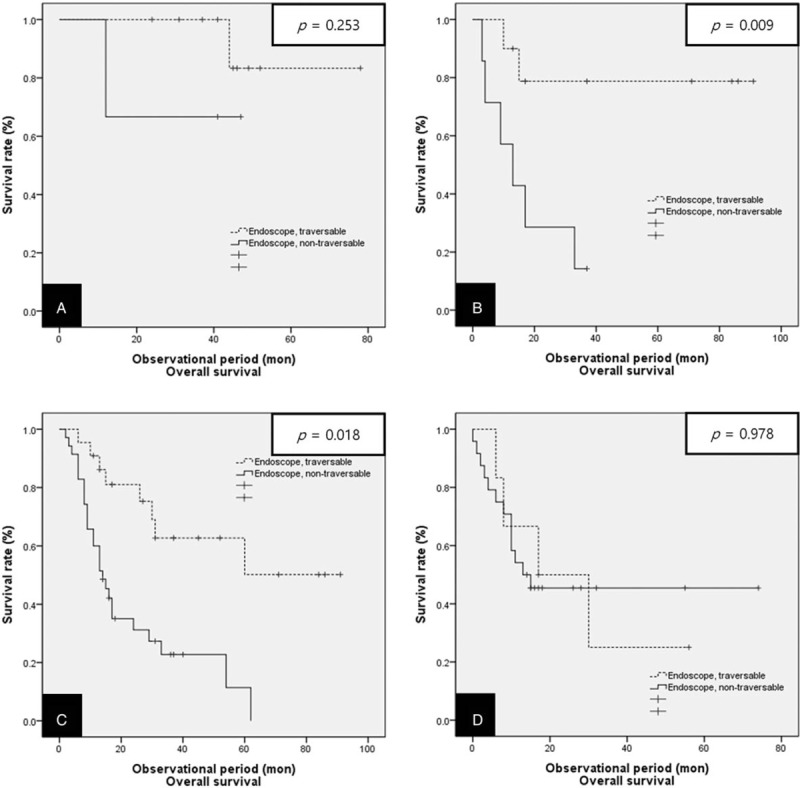
Kaplan–Meier curves for overall survival in both groups of patients treated with definitive CRT divided according to clinical stage. (A) Stage I, (B) Stage II, (C) Stage III, (D) Stage IV. CRT = chemoradiotherapy.

**Figure 6:**
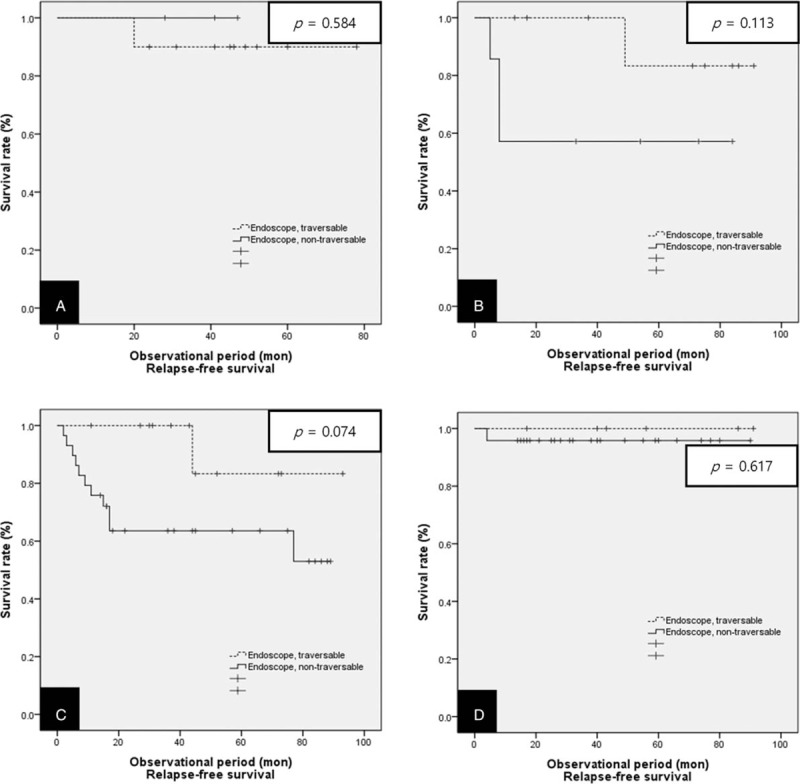
Kaplan–Meier curves for relapse-free survival in both groups of patients treated with definitive CRT divided according to clinical stage. (A) Stage I, (B) Stage II, (C) Stage III, (D) Stage IV. CRT = chemoradiotherapy.

Additionally, we performed a statistical analysis segregated according to clinical stage in terms of overall survival and relapse-free survival. For the overall survival, patients in Group A showed a significantly higher 3-year overall survival than patients in Group B only for stages II and III (50.0% vs 14.3%, *P* = .009 and 33.3% vs 17.2%, *P* = .018, respectively) (Fig. 5). However, in terms of relapse-free survival, there were no statistically significant differences according to clinical stage (Fig. 6).

### 3.4 Overall survival and relapse-free survival in patients with locally advanced esophageal cancer (stages II and III) treated with definitive CRT

**Figure 7:**
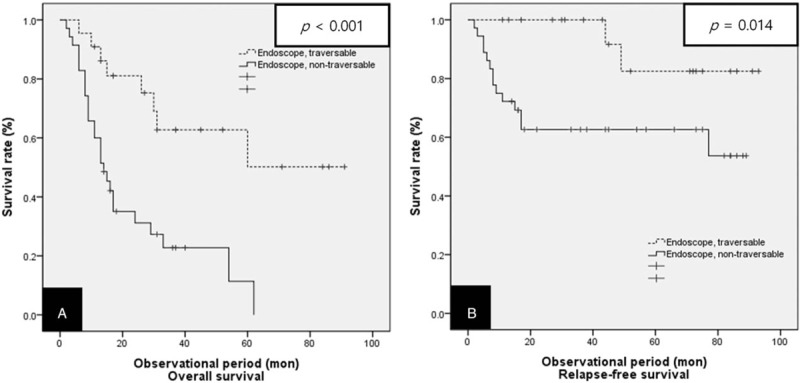
Kaplan–Meier curves for the overall survival and relapse-free survival in both groups of patients with a locally advanced stage (stages II and III) treated with definitive CRT. (A) Overall survival, (B) relapse-free survival. CRT = chemoradiotherapy.

When the 101 patients treated with definitive CRT were analyzed according to clinical stage (Figs. 4 and 5), there were no statistically significant differences in their relapse-free survival. However, for stage II and III patients treated with definitive CRT, a significantly higher 3-year overall survival was noted in Group A compared with Group B. Therefore, we performed a statistical analysis by grouping stage II and III cases classified as locally advanced esophageal cancer into a single group. Of the 58 patients with locally advanced esophageal cancer who were treated with definitive CRT, 22 (37.9%) were included in the endoscope traversable group (Group A), while the remaining 36 (62.1%) were placed in the endoscope non-traversable group (Group B). After a median follow-up of 16.33 months, the median overall survival was 60.63 months (95% CI: 44.66–76.60 months) in Group A versus 23.32 months (95% CI: 16.02–30.63 months) in Group B. Endoscope traversable patients showed a significantly higher 3-year overall survival than endoscope non-traversable patients (40.9% vs 16.7%, respectively, *P* < .001) (Fig. 7A). The median relapse-free survival time was 84.88 months (95% CI: 74.64–95.13 months) in the endoscope traversable group and only 58.01 months (95% CI: 45.17–70.85 months) for the endoscope non-traversable group. Patients in Group A showed a significantly higher 3-year relapse-free survival than Group B patients (68.2% vs 44.4%, respectively, *P* = .014) (Fig. 7B).

### 3.5 Predictive factors of survival in all patients

Univariate analysis revealed that a serum albumin level <3.5 g/dL (*P* = .001), endoscopic non-traversability (*P* = .001), advanced clinical stage (*P* = .001), and requirement for esophageal stent insertion (*P* = .001) were negative predictive factors of survival (Table 2). Similarly, a serum albumin level <3.5 g/dL (*P* = .001), endoscopic non-traversability (*P* = .007), advanced clinical stage (*P* = .033), and requirement for esophageal stent insertion (*P* = .044) were also identified as negative predictive factors of survival in multivariate analysis (Table 2).

**Table 2:**
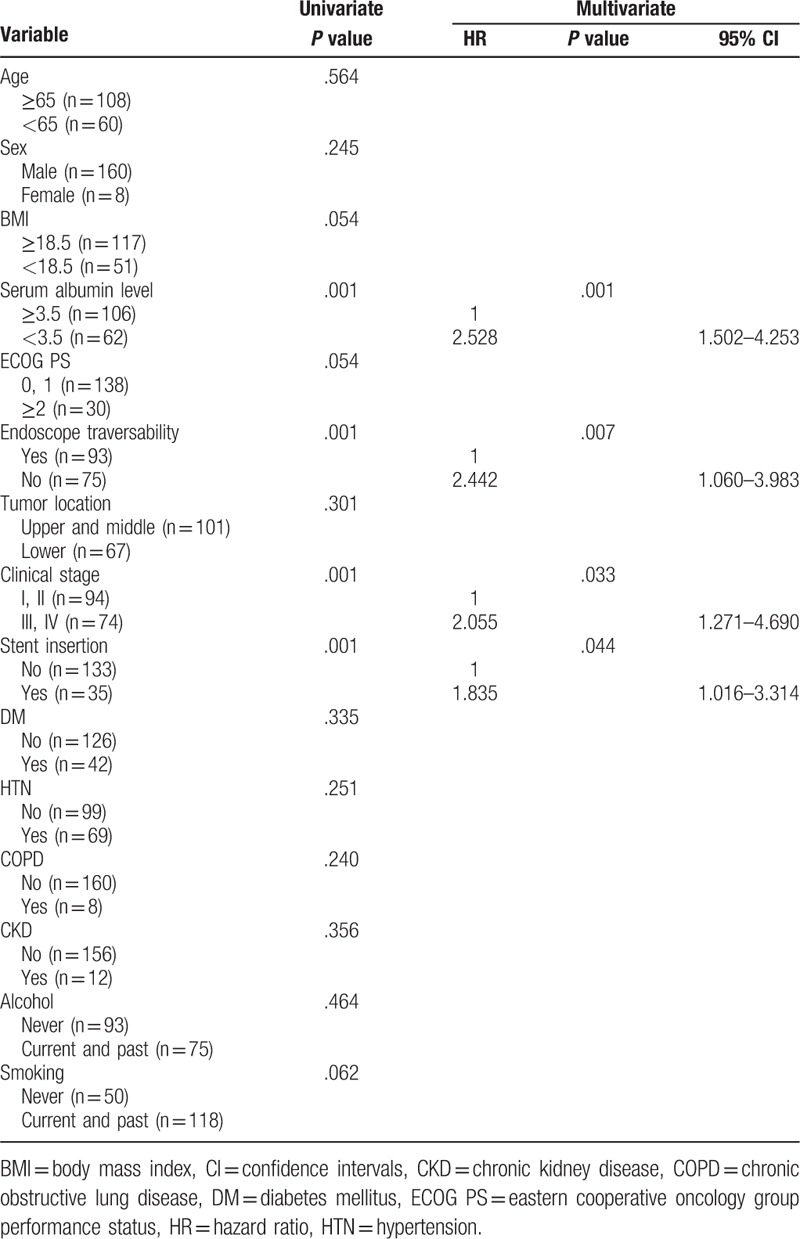
Predictive factors of survival in all patients according to univariate and multivariate analyses.

### 3.6 Predictive factors of survival in patients with locally advanced esophageal cancer (stages II and III) treated with definitive CRT

Univariate analysis revealed that a serum albumin level <3.5 g/dL (*P* = .001) and endoscopic non-traversability (*P* = .002) were negative predictive factors of survival (Table 3). Similarly, a serum albumin level <3.5 g/dL (*P* = .003) and endoscopic non-traversability (*P* = .005) were also identified as negative predictive factors of survival in multivariate analysis (Table 3).

**Table 3:**
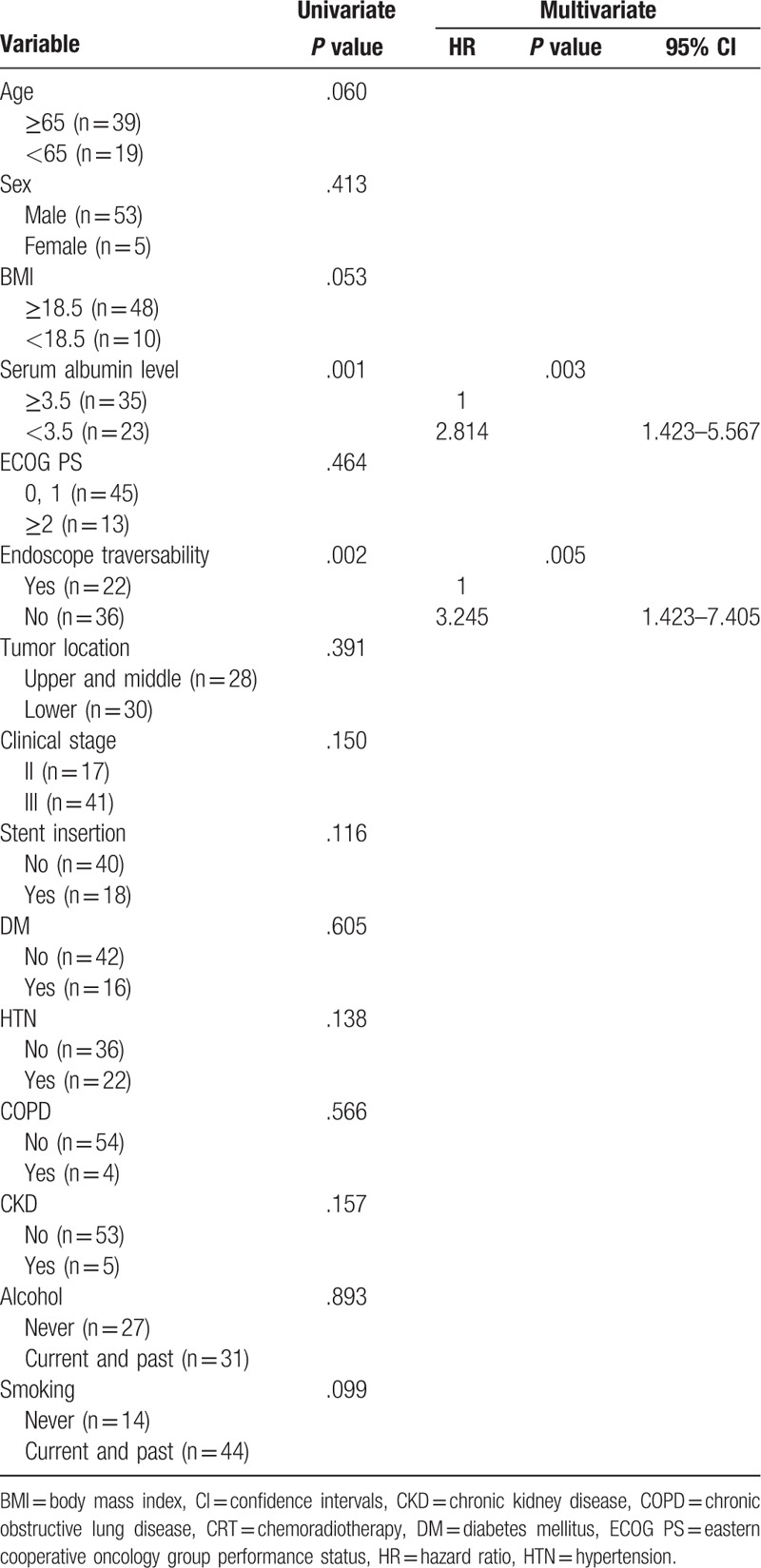
Predictive factors of survival in patients with locally advanced esophageal cancer treated with definitive CRT according to univariate and multivariate analyses.

## 4 Discussion

The results of our study showed that endoscopic non-traversability contributes significantly as a negative factor in the prognosis of patients with locally advanced esophageal squamous cell carcinoma treated with definitive CRT. Patients with endoscope traversable esophageal cancer showed a higher 3-year overall survival and 3-year relapse-free survival compared with patients with endoscope non-traversable esophageal cancer (53.8% vs 17.3%, *P* < .001 and 71.1% vs 45.3%, *P* = .003, respectively).

In locally advanced esophageal cancer, esophagectomy can be curative in a low percentage of patients. During the past 2 decades, technical developments in esophagectomy have contributed to a decrease in the morbidity and mortality of this procedure.^[9]^ These developments include more effective patient selection, advancements in preoperative staging (particularly due to EUS and 18F-fluorodeoxyglucose PET-CT), and better perioperative management and surgical skills.

To date, definitive CRT in locally advanced esophageal cancer is considered an alternative method for treatment with curative intent or as a treatment option when there are contraindications for surgery.^[10–12]^ 5-FU and cisplatin are concurrently used with radiotherapy, and they appear to have a clinically significant radiosensitizing effect.^[13]^

The poor prognosis of endoscope non-traversable esophageal cancer patients in our study can be attributed to the more advanced clinical stage of these patients; 44.6% of Group B patients had esophageal stricture. Endoscope non-traversable esophageal cancer patients also demonstrated an advanced clinical stage (*P* < .001), a poor ECOG performance status score (*P* < .001), a lower serum albumin level (3.83 ± 0.69 g/dL vs 3.44 ± 0.62 g/dL, respectively, *P* < .001), a higher rate of requirement for esophageal stent insertion (7.5% vs 37.3%, respectively, *P* < .001), and definitive CRT as their initial treatment (40.9% vs 84.0%, respectively, *P* < .001) compared with patients in Group A. The relationship between endoscopic non-traversability and an advanced stage of esophageal cancer has been previously noted. A comparison of the preoperative staging of esophageal cancer using EUS with pathologic staging of the esophagectomy specimen in 79 patients showed that 91% of patients with malignant stricture had stage III or IV disease.^[14]^ Another study involving 167 patients with esophageal cancer also reported that 88% of EUS non-traversable patients undergoing immediate surgery had T3 or T4 disease.^[15]^

Furthermore, the poorer prognosis of endoscope non-traversable esophageal cancer patients in our study may have been due to the limited EUS assessment of esophageal cancer staging. EUS is a standard staging modality for locoregional esophageal cancer and is clearly superior to CT or magnetic resonance imaging (MRI), with high T (80–90%) and N staging accuracy (70–80%).^[16]^ Nonetheless, EUS accuracy is significantly affected when the echoendoscope cannot traverse the esophageal cancer. Staging accuracy reportedly declines to 46% in EUS non-traversable esophageal cancer (vs 92% in EUS traversable esophageal cancer), while the correct preoperative T stage of patients with high-grade esophageal cancer stenosis was acquired in only 30.8% of cases (vs 81% of patients with less severe esophageal cancer stenosis).^[17,18]^ In high-grade malignant strictures, the cancer stage was determined on the basis of conventional staging work-ups. Evidence suggests that some patients with non-traversable esophageal cancer in our study who were treated with surgery or definitive CRT may have received stage-inadequate treatment, which negatively affected their survival outcomes.

The following may affect the outcome of therapy in patients with locally advanced esophageal cancer: stage of the disease; length of the tumor^[19]^; lymphatic invasion^[20]^; degree of histopathological response to the induction treatment of radiotherapy, chemotherapy, or a combination of both modalities^[21]^; performance status; possible genetic variations^[22,23]^; radiotherapy dose; additional concomitant chemotherapy for radiotherapy; histopathologic grading; hemoglobin concentration; sex; age of patients with more advanced disease^[24]^; and nutritional status.^[25,26]^ In our univariate and multivariate analysis, both endoscopic non-traversability and the serum albumin level (<3.5 g/dL) were identified as indicators of nutritional status and also predictive negative factors of survival.

Several studies have concluded that baseline nutritional status is a prognostic factor in patients with esophageal cancer who are treated with definitive CRT. Weight loss at diagnosis was identified as a prognostic factor for treatment with definitive CRT for esophageal cancer in a meta-analysis by Thomas et al^[27]^ involving 416 patients. In another retrospective study by Di Fiore et al,^[25]^ which assessed 105 esophageal cancer patients treated with definitive CRT, serum albumin levels >3.5 g/dL were an independent prognostic factor for a complete response to CRT. Furthermore, an Atkinson dysphagia score <2, an ECOG PS score <2, and a BMI >18 kg/m^2^ were all independent prognostic factors that favored overall survival. A study conducted by Wang et al^[28]^ of 123 esophageal cancer patients who received various treatment modalities showed that high C-reactive protein (CRP) and low serum albumin levels were also independent prognostic factors for survival. Clavier et al^[26]^ retrospectively analyzed the prognostic factors in 143 esophageal cancer patients treated with definitive CRT. The Nutritional Risk Index (NRI; 1.519 × serum albumin level [g/L] + 41.7 ×  [weight at the beginning of radiotherapy/baseline weight]) was an independent prognostic factor of both disease-free survival and overall survival.

For patients with malnutrition, dysphagia, and anorexia are probably the main cause. Caloric intake is often limited early in patients with esophageal carcinoma as the tumor growth obstructs the esophagus, while anorexia secondary to active catabolism by inflammatory mediators also plays a role.^[29]^ It has been previously reported that malnutrition is predictive of discontinuation of treatment as well as a poor outcome in patients treated at a palliative stage.^[30,31]^ Nutritional support has contributed to reduced weight loss, a greater radiotherapy completion rate, and fewer unplanned hospital admissions for esophageal cancer patients.^[32]^

However, our study had some limitations. First, it was a retrospective study. Therefore, the patient information might be inaccurate. Second, as all patients with esophageal squamous cell carcinoma included in this study were diagnosed and treated at our single center, there were restrictions regarding the study's general representability because of a relatively small sample size. Third, tumor stages of patients were determined not by pathologic stages, but by clinical stages using conventional imaging modalities. Finally, factors that may significantly affect survival, such as additional radiotherapy or chemotherapy, could not be considered in this study.

In conclusion, endoscopic traversability is a significant prognostic factor in patients with locally advanced esophageal squamous cell carcinoma treated with definitive CRT. This result may be due to their more advanced clinical stage, the inferior accuracy of EUS as a staging modality, and malnutrition due to malignant esophageal stricture. A strength of our study was that the prognosis of patients with esophageal squamous cell carcinoma can be easily predicted using esophagogastroduodenoscopy, which is the most essential test to diagnose esophageal cancer. Given the limitations of endoscopic traversability as a prognostic factor, additional large-scale prospective studies should be performed to determine its prognostic value.
